# Intracellular Calcium Dysregulation: Implications for Alzheimer's Disease

**DOI:** 10.1155/2016/6701324

**Published:** 2016-06-02

**Authors:** Simona Magi, Pasqualina Castaldo, Maria Loredana Macrì, Marta Maiolino, Alessandra Matteucci, Guendalina Bastioli, Santo Gratteri, Salvatore Amoroso, Vincenzo Lariccia

**Affiliations:** ^1^Department of Biomedical Sciences and Public Health, School of Medicine, University “Politecnica delle Marche”, 60126 Ancona, Italy; ^2^Department of Health Sciences, University “Magna Graecia”, 88100 Catanzaro, Italy

## Abstract

Alzheimer's Disease (AD) is a neurodegenerative disorder characterized by progressive neuronal loss. AD is associated with aberrant processing of the amyloid precursor protein, which leads to the deposition of amyloid-*β* plaques within the brain. Together with plaques deposition, the hyperphosphorylation of the microtubules associated protein tau and the formation of intraneuronal neurofibrillary tangles are a typical neuropathological feature in AD brains. Cellular dysfunctions involving specific subcellular compartments, such as mitochondria and endoplasmic reticulum (ER), are emerging as crucial players in the pathogenesis of AD, as well as increased oxidative stress and dysregulation of calcium homeostasis. Specifically, dysregulation of intracellular calcium homeostasis has been suggested as a common proximal cause of neural dysfunction in AD. Aberrant calcium signaling has been considered a phenomenon mainly related to the dysfunction of intracellular calcium stores, which can occur in both neuronal and nonneuronal cells. This review reports the most recent findings on cellular mechanisms involved in the pathogenesis of AD, with main focus on the control of calcium homeostasis at both cytosolic and mitochondrial level.

## 1. Introduction

Alzheimer's Disease (AD) is the most common type of dementia affecting millions of people. According to Alzheimer's Disease International (ADI), as of 2015 people suffering from dementia worldwide accounted for estimated 46.8 million. Approximately 70% of these cases were attributed to AD. This amount will increase to an estimated 74.7 million in 2030 and 131.5 million in 2050, with a parallel rise of healthcare costs. As a matter of fact, global costs of dementia have increased from US$ 604 billion in 2010 to US$ 818 billion in 2015, for a 35.4% increase. The incidence rate for AD grows exponentially with age, with the main onset time observed in people aged over 60, in particular between the age of 70 and 80 [1, 2]. AD has also a sex-related incidence, making women 1.5–3 times more vulnerable than men [3]. It has been widely assumed that the higher risk observed in females is related to the loss of the neuroprotective effect of sex steroid hormones during menopause, resulting in estrogen deficiency in the brain [4–6].

AD is a progressive neurodegenerative disorder leading to severe cognitive, memory, and behavioral impairment [7]. The majority of cases is idiopathic; however a rare variant of AD, known as Familial Alzheimer's Disease (FAD), accounts for a small percentage (1–5%) [2, 8] of all cases. FAD features an autosomal dominant heritability and an early disease onset (<65 years old) [7, 9]. Three genetic mutations have been identified as being responsible for FAD. They involve genes for amyloid precursor protein (APP) on chromosome 21 [10], presenilin 1 (PS1) on chromosome 14 [11], and presenilin 2 (PS2) on chromosome 1 [12]. Both forms of AD share two main pathological hallmarks: the abnormal extracellular accrual and deposition of amyloid-*β* (A*β*) peptides and the intracellular accumulation of neurofibrillary tangles (NFTs). A*β* peptides are cleaved products of APP obtained via sequential proteolysis by two membrane-bound endoproteases, aspartyl *β*-secretase and presenilin-dependent secretase (*γ*-secretase) [13, 14]. APP can also be cleaved by *α*-secretase to produce nontoxic fragments, which are thought to antagonize A*β* peptides generation [15]. A*β* is a protein consisting of 39–43 amino acids, and it mainly exists in two isoforms: soluble A*β*_1–40_ (~80–90%) and insoluble A*β*_1–42_ (~5–10%) [15, 16]. In particular, due to a greater tendency to aggregate than A*β*_1–40_, A*β*_1–42_ seems to be the main pathological isoform [17]. Interestingly, it has been described that soluble A*β* globular oligomers can form along a new aggregation pathway independent of A*β* fibril formation. These globular A*β* oligomers have been found in the brain of patients affected by AD and APP transgenic mice, and they bind specifically to neurons and affect synaptic plasticity, as demonstrated by Barghorn and coworkers [18]. The disturbance afforded by soluble A*β* oligomers has also been supported by evidence showing that they can bind to glutamate receptors (both ionotropic and metabotropic), thereby impairing glutamatergic neurotransmission [19, 20]. It is interesting to underline, however, that APP products and very low concentrations of soluble A*β* can be involved in important physiological processes, such as synapse activity and behavior [21, 22].

As for NFTs, it has been found that their major constituent is the protein tau. Tau is the predominant microtubule-associated protein found in mammalian brain [28]. During early stages of development tau is highly phosphorylated; however phosphorylation decreases with brain aging [29, 30], leading to an unphosphorylated form that binds to microtubules, thereby making them more stable. In AD, tau is aberrantly misfolded and abnormally hyperphosphorylated [7, 13]. Several factors might be involved in tau hyperphosphorylation, including A*β*-mediated caspases activation, A*β*-mediated oxidative stress, chronic oxidative stress, and reduced insulin-like growth factor 1-mediated oxidative stress [31]. Over the course of AD, hyperphosphorylation contributes to the loss of tau physiological functions and it prepares this protein to form neurotoxic aggregates. It has been shown that, in this pathological form, tau can also ectopically enter the somatodendritic compartment where, in conjunction with A*β* oligomers, it promotes excitotoxicity. Additionally, tau phosphorylation can modulate DNA integrity and global changes in transcriptional events [32].

A*β* plaques and NFTs, often referred to as “positive features” [13], occur in specific regions rather than diffusely throughout the brain: in particular hippocampus and cortex are mainly affected [8, 13]. In addition, negative features of AD have also been described, including typical losses of neurons, neuropil, and synaptic elements, that mostly parallel NFTs formation. However, a causative relationship between NFTs and neuronal loss still remains to be clarified [33–40]. Growing evidence supports the involvement of neuroinflammation in AD [41], focusing on its critical role within brain regions where A*β* plaques are mainly distributed. A*β*-deposition renders cells more likely to develop inflammatory responses that involve the production of neuronal and glial cytokines belonging to the Tumor Necrosis Factor-*α* (TNF-*α*) superfamily [42]. Interestingly, it has been shown that neutralization of the Tumor Necrosis Factor Related Apoptosis Inducing Ligand (TRAIL) protects human neurons from A*β*-induced toxicity [43]. In this context,* in vitro* experiments conducted using the differentiated human neuroblastoma cell line SH-SY5Y demonstrated that the nonsteroidal anti-inflammatory derivative CHF5074 abrogates neurotoxic effects of both A*β*_25–35_ and TRAIL [44], suggesting a potential role of this drug as neuroprotective agent.

AD patients show symptoms that can be divided into two main categories: cognitive and psychiatric. Cognitive symptoms include loss of long term memory, aphasia, apraxia, and agnosia, while psychiatric symptoms include personality changes, depression, and hallucinations (Alzheimer's Foundation of America, Last Update: January 29, 2016; [8]). AD is a complex multifactorial disorder, neuronal death is a subtle phenomenon, and it is difficult to identify a single cause. The idea that energy/mitochondrial dysfunction and oxidative stress may have a central role in the pathogenesis of AD is widely supported by literature [45–49]. Research on the pathogenesis of AD has recently stressed the role of mitochondria, based on the finding that mutation in APP and tau may directly affect mitochondrial function and dynamics [8], and now it is accepted that the impairment of mitochondrial function may affect other crucial cell signaling pathways, as in calcium signaling. A central role for calcium dysregulation in the pathogenesis of AD has been extensively suggested [7, 50]. This review attempts to clarify connections between mitochondrial pathways impairment and the pathogenesis of AD, drawing attention to the calcium homeostasis deregulation as a potential consequence of mitochondrial function disturbance and to the proteins mainly involved in this process, such as the sodium-calcium exchanger (NCX).

## 2. Calcium and AD

**Figure 1:**
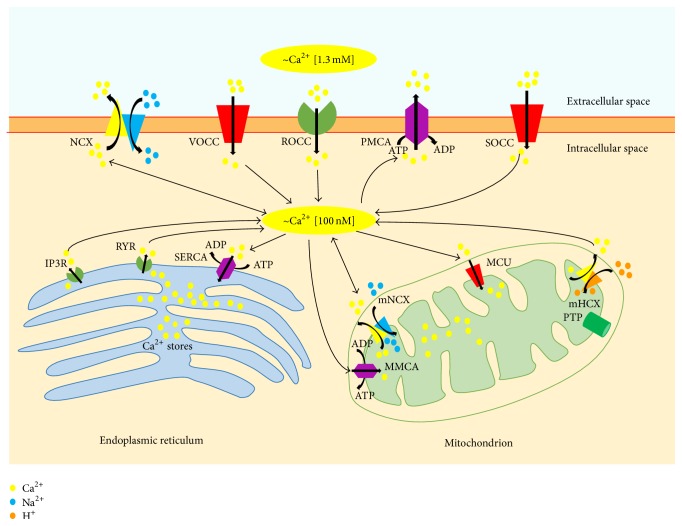
Intracellular calcium homeostasis. Intracellular calcium levels are tightly regulated within a narrow physiological range [23]. Cellular calcium influx through the plasma membrane is largely mediated by receptor-operated calcium channels (ROCC), voltage-operated calcium channels (VOCC), store-operated calcium channels (SOCC) and, under exceptional circumstances, the sodium/calcium exchanger (NCX). Under physiological conditions, NCX is mainly involved in calcium efflux; however it can also reverse its mode of operation (reverse mode exchange) thereby contributing to calcium influx, especially during strong depolarization and in the presence of high intracellular sodium concentrations [24]. Calcium may also be released into the cytoplasm from the endoplasmic reticulum, through inositol-1,4,5-trisphosphate (IP3R) and ryanodine receptors (RYR). Different systems operate within the cell to counterbalance the cytosolic calcium increase. Specifically, the plasma membrane calcium pump (PMCA), NCX, and sarcoendoplasmic reticulum calcium ATPase (SERCA) participate in restoring physiological calcium levels. The excess of intracellular calcium can also be taken up by mitochondria through the mitochondrial calcium uniporter (MCU). Calcium can be released back into the cytosol through the activity of mitochondrial NCX (mNCX), which can also reverse its mode of operation allowing the access of calcium ions into the mitochondrial matrix. Recently, the mitochondrial hydrogen/calcium exchanger (mHCX) has been proposed to be an electrogenic 1 : 1 mitochondrial calcium/hydrogen antiporter that drives the uptake of calcium into mitochondria at nanomolar cytosolic calcium concentrations [25]. PTP, permeability transition pore; MMCA, mitochondrial membrane Ca^2+^ATPase.

Calcium can be considered a ubiquitous intracellular messenger within cells acting as a regulator in multiple physiological functions. As a divalent cation, calcium can bind to several proteins, receptors, and ion channels. All of these properties are of great importance within neurons, where continuous firing of action potentials leads to calcium cycling, and it implies an influx through the calcium channels at the plasma membrane level, intracellular buffering, and an efflux through the calcium plasma membrane transporters. This cycling involves several subcellular compartments and proteins. In particular, two organelles play a major role in calcium buffering, namely, endoplasmic reticulum (ER) and mitochondria, whereas ATPase calcium pump and NCX are the two main systems involved in calcium efflux through the plasma membrane (Figure 1). Perturbation in such delicate balance may have deleterious consequences for cells and in particular for neurons, leading to necrosis and/or apoptosis and subsequently to stroke and neurodegeneration.

### 2.1. Intracellular Calcium Homeostasis

There is a large body of evidence documenting a connection between calcium homeostasis disruption and the development of neurodegenerative diseases such as Alzheimer's [50]. The involvement of calcium in the pathogenesis of AD has been suggested long time ago by Khachaturian [51], and since then many efforts have been made to clarify this hypothesis [7, 52–56]. Despite the significant progresses made in explaining this theory, several aspects are to be defined. For instance, growing* in vitro* evidence suggests that neuroprotection could be mediated by the restoration of calcium homeostasis. Different calcium channel blockers have been reported to be effective in preventing long- and short-term memory impairment induced by A*β*_25–35_ (the shortest A*β* fragment processed* in vivo* by brain proteases, retaining the toxicity of the full-length peptide [57]) and in decreasing A*β* production, inflammation, and oxidative stress. For example, Rani et al. described the effect of a calcium channel blocker clinically used in angina, in a mouse model of dementia. Interestingly, Morris water maze test, plus maze test and different biochemical analysis, demonstrated the restoration of normal learning and memory functions. Moreover, SCR-1693 (a nonselective calcium channel blocker) has been described to attenuate A*β*_25–35_-induced death in SH-SY5Y cells and to regulate A*β*-induced signal cascade in neurons [58–60]. However, the use of calcium channel blockers to mitigate AD outcomes is still much debated. For example, at least three clinical studies emphasized that elderly people, taking calcium channel blockers as antihypertensive drugs, were significantly more likely to experience cognitive decline than those using other agents [61–63].

At cellular level, it is well documented that abnormal amyloid metabolism induces an upregulation of neuronal calcium signaling, firstly resulting in a decline of memory and then leading to apoptosis [7, 50, 51, 64, 65]. An interesting connection between A*β*, calcium, and AD has been postulated by Arispe and coworkers [66], who suggested that A*β* oligomers can form calcium-permeable channels in membranes. It seems that energy deficits can promote this association, consistently with the observation that neurons with low cytosolic ATP levels showed a pronounced vulnerability to A*β*-induced toxicity [67]. In line with these reports, studies conducted in animal models (i.e., transgenic mice) highlighted an increase in calcium resting levels in the spines and dendrites of pyramidal cortical neurons [68, 69], supporting the hypothesis that calcium-permeable channels can form in the neuronal plasma membrane close to the A*β* plaques, thanks to the high concentration of A*β* oligomers found in these areas [67]. Tau protein is also able to form ion channels in planar lipid bilayer, with lack of ion selectivity and multiple channels conductance, thus contributing to lower membrane potential, dysregulate calcium, depolarize mitochondria, or deplete energy stores [70]. Within neurons, the increase in intracellular calcium levels stimulated by A*β* does not seem to be necessarily sustained by extracellular calcium influx. By using the human neuroblastoma SH-SY5Y cell line, Jensen and coworkers [71] interestingly described that the increase in intracellular calcium levels elicited by the A*β*_1–42_ fragment can occur in the absence of extracellular calcium. Such observation supports the role of calcium release from the ER [72] to the generation of these signals. In addition, they demonstrated that this phenomenon relies only partially on inositol 1,4,5-trisphosphate (IP3) signaling, based on the fact that they observed the calcium mobilizing effect of A*β*_1–42_ when the fragment was applied to permeabilized cells deficient in IP3 receptors (IP3R). Notably, this effect could underpin an additional direct effect of A*β*_1–42_ upon the ER and a mechanism for induction of toxicity by intracellular A*β*_1–42_ [71]. As a matter of fact, ryanodine receptors (RyR) can also contribute to the A*β*-induced calcium release from ER, as described by Ferreiro and coworkers [73, 74]. Exposing rat primary cortical neurons to A*β*_1–40_ or to A*β*_25–35_ peptides, the authors observed an increase in cytosolic calcium levels that was counteracted by either xestospongin C or dantrolene, pharmacological inhibitors of IP3R and RyR, respectively. Once calcium has been mobilized, it can initiate a cascade of events promoting free radicals generation, cytochrome c release from mitochondria, and activation of caspases, culminating in apoptotic cell death [73, 74]. It is worth mentioning that the balance between intracellular calcium levels and ER content involves not only IP3R and RyR, but also the activity of sarcoendoplasmic reticulum calcium ATPase (SERCA), which transports calcium ions from the cytoplasm into the ER (Figure 1). In this regard, Ferreiro and coworkers performed a comparative study by using the selective SERCA blocker thapsigargin [74]. They demonstrated that thapsigargin induced the loss of intracellular calcium homeostasis and the activation of caspase-3, leading to apoptotic cell death, as observed after incubation with A*β*_1–40_ or A*β*_25–35_ peptides. These findings lend support to the hypothesis that intracellular calcium deregulation induced by ER stress may be critical in the neurodegenerative processes triggered by A*β* peptide. Furthermore, the role of SERCA has been also investigated in the context of the FAD. Specifically, it has been proposed that SERCA activity is physiologically regulated by the interaction with presenilin [75], the membrane intrinsic protein that localizes predominantly to the ER membrane, which is responsible for the generation of the A*β* fragment. The finding that the modulation of SERCA activity would alter A*β* production may entail a possible role of the SERCA in the pathogenesis of AD [76].

The alteration of the glutamatergic system may be another important factor causing calcium imbalance in AD. Once released at glutamatergic synapses, glutamate is cleared from the extracellular space by the activity of the high affinity sodium-dependent glutamate transporters (Excitatory Amino Acid Transporters, EAATs) [77], which represent the most prominent system involved in terminating the excitatory signal, recycling the transmitter, and regulating extracellular levels of glutamate. As a result of overproduction and/or impaired clearance from synapses, glutamate may become excitotoxic. In this case, a prolonged exposure to glutamate induces an excessive activation of glutamate receptors, which is associated with a massive calcium influx through the receptor's associated ion channel. The resulting calcium overload is particularly neurotoxic, leading to the activation of several degradation pathways which can have deleterious consequences on the cell fate [78–80]. Marked changes in functional elements of the glutamatergic synapses, such as glutamatergic receptors and transporters, have been described in AD. In 1996, Masliah and coworkers observed a deficit in glutamate transport activity in AD brains, likely occurring at neuronal level [81]. In line with this report, more recent findings suggested that soluble A*β* oligomers can disrupt neuronal glutamate uptake and promote long-term synaptic depression (LTD), a form of synaptic plasticity. In particular, the elegant study by Li and coworkers [82] showed that soluble A*β* oligomers from several sources, including human brain extracts, facilitated electrically evoked LTD in the mouse hippocampal CA1 region, involving both metabotropic and ionotropic glutamate receptors, and high extracellular glutamate levels. Accordingly, neuronal synaptic glutamate uptake was significantly decreased by A*β*. It is interesting to note that A*β*-facilitated LTD was mimicked by the action of the glutamate reuptake inhibitor DL-threo-beta-benzyloxyaspartate (TBOA), confirming that A*β* oligomers ability to perturb synaptic plasticity may rely upon glutamate recycling alteration at the synaptic level. In this regard, a dramatic reduction in the expression of two members of the EAAT family, EAAT1 and EAAT2, has been described at both gene and protein levels in hippocampus and gyrus frontalis medialis of AD patients [83]. Interestingly, in the same regions, glutamate receptors of the kainate type were significantly upregulated, further supporting the hypothesis that excitotoxic mechanisms can have a role in the pathogenesis of AD [79]. Such upregulation was accompanied by downregulation of the other ionotropic glutamate receptors, namely, N-methyl-D-aspartate (NMDA) and *α*-amino-3-hydroxyl-5-methyl-4-isoxazole-propionate (AMPA) receptors. Considering that both NMDA and AMPA receptors are known to mediate long-term potentiation [84, 85], the fundamental molecular mechanism of learning, memory, and cognition, their impairment may be considered a causative factor of the reduced cognitive functions observed in AD patients [83].

Although the observed alterations in intracellular calcium homeostasis in neurons significantly contribute to the pathogenesis of AD, more recent findings suggest that calcium dysregulation occurring in other cell types that support neuronal activity may contribute to degenerative processes [86]. In this regard, Fonseca and colleagues have recently demonstrated that A*β* may imbalance calcium homeostasis in brain endothelial cells with an increase in oxidative stress [87]. Using rat brain microvascular endothelial cells, they showed that the exposure to a toxic dose of A*β* alters ER ability to buffer calcium, and it enhances the mitochondrial and cytosolic response to ATP-stimulated ER calcium release. Although these responses are compensated after a longer exposure to A*β*, the early increase in oxidant levels and the concomitant decrease of antioxidant defenses induce deleterious effects on endothelial cells that undergo apoptosis, contributing to the cerebrovascular impairment observed in AD [87].

Astrocytes are also emerging as active players in AD [88], as highlighted in a recent paper by Dal Prà and coworkers [89]. They suggested an interesting issue concerning A*β* interaction with the Calcium Sensing Receptor (CaSR) [90]. The CaSR is a member of the largest family of cell surface receptors, the G protein-coupled receptors involved in calcium homeostasis. CaSRs expression is ubiquitous within the brain [91], where they are involved in several physiological processes, including synaptic plasticity and neurotransmission [92]. They showed that, in astrocytes, CaSR-A*β* interaction induces a downregulation of CaSR, leading the neighboring neurons to oversecrete* de novo* synthesized A*β* as well as nitric oxide (NO) and the toxic peroxynitrite (ONOO^−^) [90, 93]. Recently, they have shown that the interaction occurring between A*β* and CaSR in human astrocytes may activate a signaling able to stimulate* de novo* production and secretion of vascular endothelial growth factor (VEGF) [89], whose excessive production can have toxic effects on neurons, astrocytes, and brain–blood barrier [94–97].

In general, the available literature suggests that the prolonged intracellular calcium elevation occurring within brain cells may be a crucial early event in AD pathogenesis, even though the mechanisms have not been fully explained.

In terms of proteins contributing to the calcium homeostasis in the brain, particular attention should be focused on NCX. NCX is a transporter that can move sodium across the membrane in exchange for calcium, operating in either calcium-efflux/sodium-influx mode (forward mode) or calcium-influx/sodium-efflux mode (reverse mode) depending upon the electrochemical ion gradients [24]. Three NCX isoforms have been described, namely, NCX1, NCX2, and NCX3, whose pattern of expression is tissue-specific [98]. Recent reports demonstrated the main role of NCX1 in controlling energy metabolism in several cells types, including neurons and astrocytes [99, 100]. In detail, our group recently reported a functional interaction between NCX1 and the sodium-dependent Excitatory Amino Acid Carrier 1 (EAAC1), at both plasma membrane and mitochondrial level in neuronal, glial, and cardiac models [99, 100]. Notably, we found that NCX1 reverse activity is necessary to restore transmembrane sodium gradient after glutamate entry into the cytoplasm, supporting glutamate utilization as a metabolic substrate that, in turn, enhances ATP production.

The role of NCX isoforms in the pathogenesis of AD is still under investigation. In 1991 Colvin and coworkers [101], measuring NCX activity in cerebral plasma membrane vesicles purified from human postmortem brain tissues of normal, AD, and non-AD origin dementia, identified a transporter altered kinetic in the vesicles of AD patients. The surviving neurons showed an increased NCX activity, leading authors to speculate that this phenomenon could help the surviving neurons to overtake the neurodegenerative process of AD, reinforcing the idea that the increase in intracellular calcium levels can play a major role in the pathogenesis of AD entailing the death of nonsurviving neurons. The hypothesis of an altered activity of NCX in AD patients represents an attractive mechanism that could, at least partially, be accountable for the calcium dysregulation observed in neurodegenerative processes accompanying the pathology [102]. The impairment of NCX activity can be related to the main features of AD. For instance, aggregated A*β* could interact with the hydrophobic surface of NCX, leading to an altered activity of the transporter [103]; however, it cannot be excluded that the observed interaction of A*β* oligomers with the plasma membrane could be* per se* responsible for the alteration of NCX transport properties [103]. The pioneering study of Colvin has inspired further studies that explained the specific role of different NCX isoforms in AD; in this regard, the study by Sokolow and coworkers offered a better understanding of the actual role of NCXs [104]. The analysis of NCX1, NCX2, and NCX3 expression in AD parietal cortex disclosed a specific pattern of expression within nerve terminals. In particular, NCX1 is the main isoform expressed in nerve terminals of cognitively normal patients, while NCX2 and NCX3 seem to be modulated in the parietal cortex in a late AD stage, as NCX2 expression is increased in positive terminals, while NCX3 expression is reduced [104]. Interestingly, the three isoforms colocalize with A*β*, supporting the hypothesis that the NCX activity modulation can be connected to a direct interaction with A*β*; furthermore, in all synaptic terminals containing A*β*, NCX1-3 expression is upregulated [104]. It could be possible that the altered expression of NCX isoforms represents the neurons attempt to counterbalance the A*β*-induced alteration in calcium homeostasis. But, the different pattern observed in NCX isoforms expression can underpin a specific role for each isoform within the neurodegenerative process accompanying AD. In this regard, a specific alteration has been demonstrated for NCX3 isoform, leading to inactivation. NCX proteins can be inactivated by specific calpain 1 operated cleavage, and this can produce an increase of intracellular calcium levels contributing to the neurodegenerative calcium overload [105, 106]. In AD, the overproduction of A*β* increases calpain-mediated cleavage of NCX3, resulting in a decreased NCX3 activity [107]. Interestingly, the localization of NCX3 in dendrites and astrocytes processes contacting excitatory synapses [108] suggests the major role of NCX3 in regulating calcium current during synaptic activity, which is crucial for normal learning and memory. Therefore, reduced NCX3 activity can strongly contribute to the altered calcium levels associated with neuronal dysfunctions in AD [107].

## 3. Mitochondria and AD

Mitochondria are essential organelles for both cell survival and death, as they produce the largest part of cellular energy in the form of ATP and they play an active role in apoptosis induction [109, 110]. Mitochondria take part in cellular calcium signaling and act as highly localized buffers, thereby acting in the regulation of cytosolic calcium transient [111–113] (Figure 1). A crucial role in neurodegenerative disorders has been suggested for mitochondria, and AD patients have shown evidence of impaired mitochondrial function [114]. Reddy and coworkers demonstrated the upregulation of genes related to mitochondrial energy metabolism and apoptosis in an AD transgenic mouse model overexpressing a mutant form of APP at different stages of AD progression [115]. Mutant APP and soluble A*β* may enter mitochondria, which generate reactive oxygen species leading to oxidative damage, thereby affecting mitochondrial function. That is why the upregulation of mitochondrial genes could be a compensatory response to mitochondrial dysfunction induced by mutant APP or A*β* [115, 116].

In healthy neurons synaptic activity can be influenced by mitochondrial dynamics, such as fission and fusion events [117]. A number of studies demonstrate that essential proteins for fission and fusion are altered when APP is overexpressed [118, 119]. It has been shown that dynamin-like protein 1 (DLP1) and optic atrophy (OPA1) protein are significantly decreased, whereas levels of fission 1 (Fis1) are significantly increased in cell lines overexpressing APP [119]; this leads to mitochondrial fragmentation and abnormal distribution, which contribute to mitochondrial and neuronal dysfunction [119]. These findings were confirmed by Gan and coworkers [120] that observed significant changes in mitochondria morphology and function in cytoplasmic hybrid (cybrid) neurons, where platelet mitochondria from AD and non-AD human subjects were incorporated into mitochondrial DNA-depleted neuronal cells. They found an impairment of fission/fusion proteins expression and function that was reverted by antioxidant treatment. Interestingly, they showed that oxidative stress negatively affects the extracellular-signal-regulated kinases (ERK) transduction pathway, which alters the expression levels of mitochondrial fission/fusion protein in AD cybrids [120].

Although it was common to focus primarily on A*β*, recently there has been an increasing interest on the role of the hyperphosphorylated form of tau. Hyperphosphorylation can decrease tau binding to microtubules, thereby affecting their stability and axonal transport of organelles, including mitochondria [8, 31]. Recent studies have begun to explore the effect of this altered protein on mitochondrial dynamics. Interesting findings come from the experiments performed by Schulz and coworkers [121] in SH-SY5Y wild-type (wt) and overexpressing P301L mutant tau. They demonstrated that P301L overexpression results in a substantial complex I deficit accompanied by decreased ATP levels and increased vulnerability to oxidative stress. Interestingly, those events were paralleled by pronounced changes in mitochondrial morphology and decreased fusion/fission rates, observed as reduced expression of several fission and fusion proteins such as OPA-1 or DLP- 1 [121]. An imbalance in fission/fusion proteins has also been shown by Manczak and Reddy [122] who demonstrated a physical link between phosphorylated tau and DLP1. The authors concluded that the interaction between phosphorylated tau, DLP1, and A*β* can cause an excessive mitochondrial fragmentation and both mitochondrial and synaptic deficiencies, leading to neuronal damage and cognitive decline [122]. Regardless of its connection with fission/fusion events, the synergistic action of A*β* and tau has been further investigated in a recent study by Quintanilla and colleagues who demonstrated that, in aging neuronal cultures, phosphorylated tau potentiates A*β*-induced mitochondrial dysfunction by affecting mitochondrial membrane potential and increasing oxidative stress [123]. In a previous study, the same group demonstrated that also a truncated form of tau, cleaved at Asp421 by caspases [124], significantly increases oxidative stress response in cortical neurons treated with sublethal concentrations of A*β* [125]. Moreover, interesting results in this field have been obtained by using triple transgenic mice. This model has been obtained by cross-breeding tau transgenic pR5 mice, characterized by tangle formation, and double-transgenic APP152 mice developing A*β* plaques. Only triple transgenic mice, combining both pathologies, at early age (8 months old) showed a reduction of the mitochondrial membrane potential, while at the age of 12 months they showed the strongest defects on oxidative phosphorylation, synthesis of ATP, and reactive oxygen species formation, emphasizing synergistic and age-associated effects of A*β* and tau in perishing mitochondria [126]. Globally, these findings clearly demonstrate that mitochondrial function can be seriously impaired by A*β* and that hyperphosphorylation of tau can enhance the A*β*-induced mitochondrial neuronal damage. Notably, mitochondria are also involved in the maintenance of cellular activities through the contact they establish with ER [127, 128]. Mitochondria-associated ER membranes (MAMs) are intracellular lipid rafts regulating calcium homeostasis and several metabolic pathways, such as glucose, phospholipids, and cholesterol metabolism [127, 129]. The physical interaction between these organelles has been extensively studied, and several MAMs-associated proteins have been identified. A recent research has shown that the contact sites between mitochondria and ER are enriched in PS1 and PS2 [130], components of the *γ*-secretase complex which processes APP to produce A*β* [131]. A large body of evidence indicates PS1 and PS2 mutations as being responsible for the A*β* overproduction by *γ*-secretase activity leading to FAD [132, 133]. Recently, it has been shown that mutations in PS1, PS2, and APP can upregulate MAMs function and produce a significant increase in ER-mitochondrial connectivity, suggesting that presenilins can negatively regulate this phenomenon [134]. However, the same upregulation in MAMs function and ER-mitochondrial communication has been found in fibroblasts from patients with sporadic AD (SAD), in which there are no mutations in PS1, PS2, and APP structure [134]. This interesting finding suggests that the upregulated function of MAMs, as a common feature in both FAD and SAD, may represent a pathogenic initiator of AD [134]. A recent study by Schreiner and colleagues [135] supports this hypothesis. In this work, the authors determined the production of A*β* in subcellular fractions isolated from mouse brain. They found that a large amount of A*β* was produced at mitochondria-ER contact sites. They postulated that the enhanced A*β* production may perturb mitochondria and mitochondria-ER contact site functions, leading to neurodegeneration and, therefore, to AD [135]. As a matter of fact, the MAMs structure has been postulated to modulate calcium signals and synaptic and integrative activities at neuronal level [127, 136]. In this regard, it has been suggested that MAMs may host important physiological functions related to neuronal integrity, as they have been reported to be uniformly distributed throughout hippocampal neurons and at synaptic level [127]. In particular, two main proteins have been identified as being crucial for MAMs activity and, consequently, for neuronal integrity: phosphofurin acidic cluster sorting protein-2 (PACS-2) and *σ*1 receptor (*σ*1R) [127]. These proteins contribute to maintaining MAMs homeostasis. Specifically, PACS-2 is a multifunctional sorting protein controlling ER-mitochondria communication and apoptosis [137], whereas *σ*1R promotes calcium transport into mitochondria from the ER by interacting with the IP3R [138]. Their knockdown results in neurodegeneration, and this highlights the importance of these proteins in the maintenance of neuronal integrity [127]. Furthermore, exposure to A*β* results in the increase of MAMs-associated proteins expression and of the amount of contact points between ER and mitochondria in different AD models (namely, APP transgenic mice, primary neurons, and AD brain) [127]. In turn, the alteration in MAMs-associated proteins expression can affect calcium homeostasis, which has been considered an underlying and integral component of AD pathology [7, 50, 67]. This issue is further discussed in the following section.

### 3.1. Role of Mitochondria in Intracellular Calcium Balance

**Figure 2:**
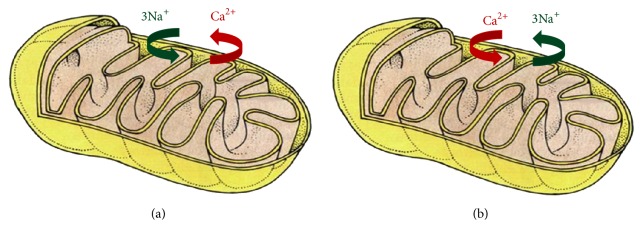
Modes of operation of mNCX. The figure reports the prevalent modes of operation of mNCX. (a) shows the* forward mode* of operation of the exchanger, which is prevalent in physiological conditions. In this mode of operation, mNCX mediates the extrusion of calcium ions from mitochondrial matrix in exchange for sodium ions. (b) shows mNCX* reverse mode* of operation. In this mode of operation, the mitochondrial exchanger mediates the influx of calcium ions into the matrix and the extrusion of sodium ions. The figure has been entirely reproduced from Castaldo et al., 2009 [26], upon written authorization by the editor.

**Figure 3:**
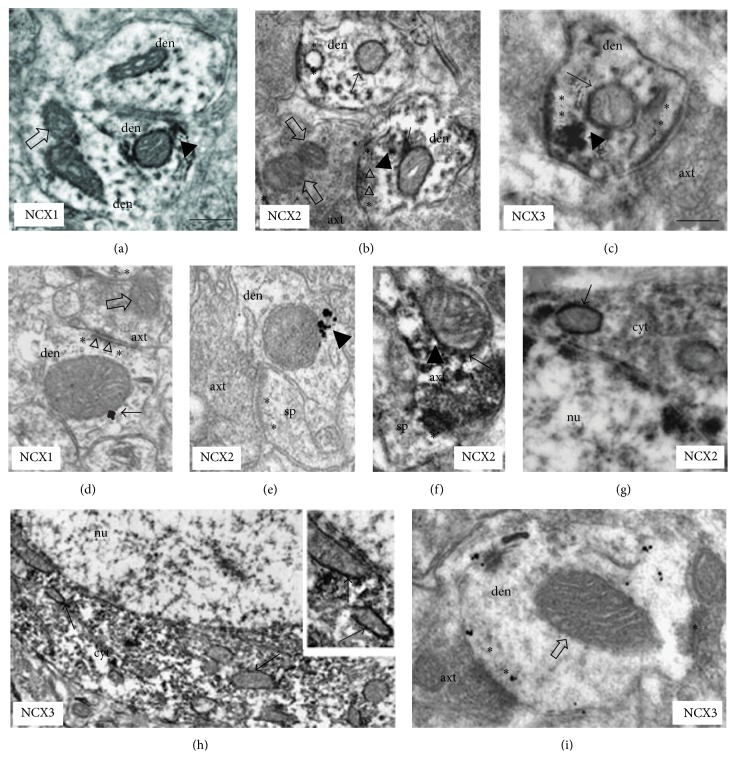
NCXs labeling patterns in neuronal mitochondria ((a)–(i)). (a and d) NCX1-ir mitochondria (arrows) in distal dendrites (CA1 stratum radiatum). ((b) and (e)) NCX2-ir mitochondria (arrows) in hippocampal (b) and neocortical (e) dendrites. (c) NCX3-ir mitochondria in neocortical distal dendrite (arrow). (f) NCX2-positive mitochondrion in a CA1 axon terminal. ((g) and (h)) NCX2 and NCX3-ir mitochondria (arrows) in a cell body (from CA1 pyramidal cell layer); enlarged in the inset in (h), two labeled organelles (arrows) near nuclear envelope. (i) NCX3-ir in neocortical distal dendrite with unlabeled mitochondria (open arrow). In (b) and (d) dendrites are contacted by axon terminals forming asymmetric junction (triangles). In (a) and (e), note the labeling bridging plasma membrane and mitochondrial profile. Open arrows indicate unlabeled mitochondria in dendrites ((a) and (i)) and axon terminals ((b) and (d)). With asterisks the postsynaptic specializations are indicated and the arrowheads show the labeling between mitochondria and plasma membrane. axt, axon terminal; den, dendrite; nu, nucleus; cyt, cytoplasm; sp, dendritic spine. Immunoperoxidase reaction in (a)–(c), (f), (g), and (h) and silver-enhanced immunogold in (d), (e), and (i). Calibration bars: in (a), 0.25 m for (a), (b), (d), (f), and (i); in (a), 0.5 m for inset in (h); in (c), 0.25 m for (c) and (e); in (c), 0.5 m for (g); in (c), 1 m for (h). The figure has been entirely reproduced from Gobbi et al., 2007 [27], upon written authorization by the editor.

**Figure 4:**
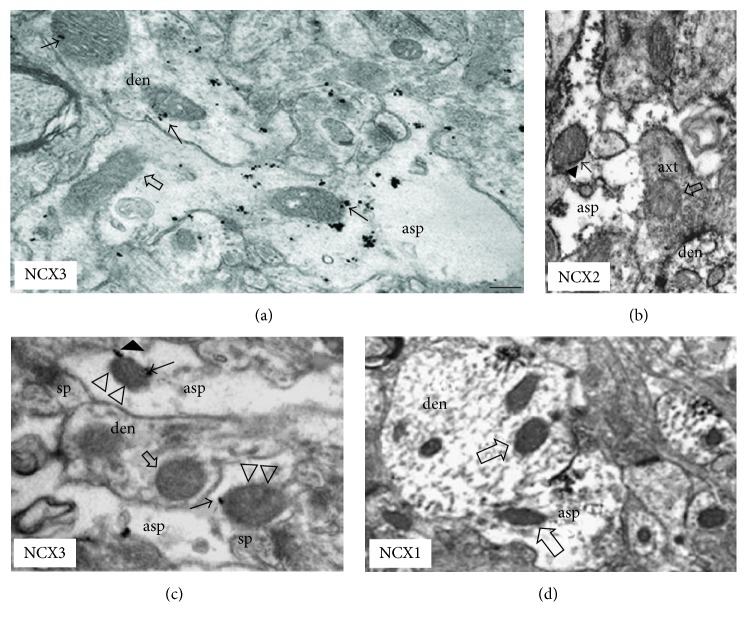
NCXs labeling patterns in astrocytic mitochondria ((a)–(d)). (a) NCX3-expressing mitochondrion (arrow) in neocortical astrocytic process; an adjacent dendrite contains two labeled mitochondria (arrows). (b) NCX2-ir mitochondrion (arrow) in hippocampal glial process. Intense labeling is present on plasma membrane. (c) NCX3-labelled sub-plasma membrane mitochondria (arrows) in two astrocytic processes contacting synaptic structures; labeling between a mitochondrion and the plasma membrane is evident (arrowhead). An unlabeled mitochondrion is localized in a dentritic structure (open arrow). (d) A NCX1-unlabeled mitochondrion (open arrow) in a labeled distal astrocytic process in neocortex. Note some positive distal dendrites with unlabeled mitochondria. Open arrows indicate unlabeled mitochondria; triangles show the mitochondrial labeling near the synaptic membrane. Asp, astrocytic process; den, dendrite; axt, axon terminal; sp, spine apparatus. Immunoperoxidase reaction in (b) and (c) and silver-enhanced immunogold in (a). Calibration bars: in (a), 0.25 m for (a), (b), and (c); in (a), 0.5 m for (d). The figure has been entirely reproduced from Gobbi et al., 2007 [27], upon written authorization by the editor.

Intracellular calcium dysregulation is a central event in neurodegeneration; it involves plasma membrane transporters and also intracellular organelles, such as mitochondria, thereby creating an endless futile cycle that can have several consequences on neuronal survival [67]. The excess of intracellular calcium is taken up by mitochondrial calcium uniporter (MCU) that, through the large electrochemical gradient across the inner mitochondrial membrane, drives calcium from the cytosol to the mitochondrial matrix [67] (Figure 1). Calcium is then released back into the cytosol through the activity of mitochondrial NCX (mNCX, Figures 1 and 2(a)) [67]. However, mNCX may reverse its mode of operation (Figure 2(b)) from a calcium efflux system to an influx pathway allowing the access of calcium ions into the mitochondrial matrix [26]. Although the molecular identity of mNCX has been extensively researched and strongly debated, our group has provided data showing that plasma membrane NCX (plmNCX) isoforms can contribute to mNCXs. Exploring the subcellular distribution of NCX in the central nervous system by western blot and* in situ* electron microscopy immunocytochemistry in rat neocortex and hippocampus, we observed a large population of neuronal and astrocytic mitochondria expressing NCX1–3 [26, 27] (Figures 3 and 4). Thus, these mitochondrial calcium transporters manage intracellular changes of this “versatile” ion, impacting several cell functions, including cell metabolism. As a matter of fact, the activity of several intramitochondrial dehydrogenases is enhanced by increased mitochondrial calcium levels, thereby stimulating ATP synthesis [139, 140]. The brain is one of the most metabolically active organs in the body. The brain's high energy requirements are mainly due to maintenance and restoration of ion gradients dissipated by signaling processes such as postsynaptic and action potentials, as well as uptake and recycling of neurotransmitters. In AD, the impairment in energy production is one of the factors greatly contributing to the vulnerability of neuronal cells [141]. One of the main works demonstrating the cooperative action of tau and A*β* shows, through a proteomic analysis, that one-third of the deregulated proteins in different AD mouse models is made up of mitochondrial proteins involved in oxidative phosphorylation [126]. Hence, it is tempting to speculate that modulation of mitochondrial calcium transporter activity toward the increase in ATP production could have beneficial effects on neuronal survival during the neurodegenerative processes that characterize AD. In this context, it has been suggested that a partial inhibition of mNCX would lead to an increase of the mitochondrial matrix calcium concentration to a higher physiological steady-state level that could stimulate calcium-sensitive dehydrogenase activity and the rate of ATP synthesis [67, 139, 140]. Therefore, calcium may play a dual role within cells: on the one hand it can help vulnerable neurons increase the rate of ATP synthesis; on the other hand it can be harmful and activate cell death through the induction of the apoptotic pathways [142]. Thus, there must be a critical point representing the boundary between cytoprotective and cytotoxic effect due to the increase in mitochondrial calcium concentration [67]. An increased rate of ATP synthesis can be achieved stimulating the cell in several ways. Recently, our group found that both plmNCX and mNCX can act synergically to sustain the increase in ATP synthesis promoted by glutamate [99, 100]. As reported above, this metabolic response results from a physical and functional interaction between NCX (particularly NCX1) and EAATs, with particular reference to EAAC1, occurring at both plasma membrane and mitochondrial level [99, 100]. The fact that some substrates, such as glutamate, can modulate ATP synthesis may have several implications for AD too, and this can reverse the traditional view of a predominantly harmful effect of this amino acid, towards a benefic role that is able to rescue vulnerable neurons from death. At present, the role of mNCX in AD is still largely unexplored [26]. In an interesting paper, Thiffault and Bennett [143] reported indirect evidence of an involvement of the exchanger in AD. In particular, they showed that cells, lacking endogenous mitochondria and repopulated with mitochondria from AD patients, virtually lack the spontaneous fluctuations in mitochondrial membrane potential (ΔΨ_M_), also called “ΔΨ_M_ flickering,” which is normally induced by cyclosporine. It is worth noting that mNCX blockade with CGP-37157 suppresses flickering in control cells, thus recreating a condition similar to the one observed in AD. The role of mNCX in AD is also supported by the work of Chin and colleagues [144], who observed that A*β* potentiates the increase in cytosolic calcium concentration evoked by nicotine in dissociated rat basal forebrain neurons in a CGP-37157-sensitive way.

## 4. Conclusions

Dysregulation of intracellular calcium homeostasis has been suggested as a proximal cause of cellular dysfunction during AD, and in this context calcium imbalance has been considered a phenomenon mainly related to the dysfunction of subcellular organelles, such as mitochondria. Functional impairment of calcium-related proteins may play a major role in the pathogenesis of AD. One of the main regulators of intracellular calcium levels, NCX, is emerging as a transporter possibly involved in the nervous system pathophysiology, although its involvement in AD is still poorly investigated. Recent studies conducted by our group [99, 100] show NCX as a key factor in the regulation of cellular metabolism too, acting at both plasma membrane and mitochondrial level. Energy metabolism and intracellular calcium levels are closely related and, therefore, it has been suggested that energy and calcium signaling deficits can be considered the earliest modifiable defects in brain aging [145], including AD. The achievement of an increase in cell metabolism and mitochondrial calcium content through the manipulation of NCX activity may represent a new successful approach to prevent neuronal degeneration and death. However, further studies are needed to support this finding.
